# Optimizing moving average control procedures for small-volume laboratories: can it be done?

**DOI:** 10.11613/BM.2019.030710

**Published:** 2019-10-15

**Authors:** Vera Lukić, Svetlana Ignjatović

**Affiliations:** 1 Department of laboratory diagnostics, Railway Healthcare Institute, Belgrade, Serbia; 2 Department of Medical Biochemistry, University of Belgrade, Faculty of Pharmacy, Belgrade, Serbia; 3 Center for Medical Biochemistry, Clinical Center of Serbia, Belgrade, Serbia

**Keywords:** quality control, moving average, bias detection simulation, MA Generator software

## Abstract

**Introduction:**

Moving average (MA) means calculating the average value from a set of patient results and further using that value for analytical quality control purposes. The aim of this study was to examine whether the selection, optimization and validation of MA procedures can be performed using the already described bias detection simulation method and whether it is possible to select appropriate MA procedures for a laboratory with a small daily testing volume.

**Materials and methods:**

The study was done on four analytes: creatinine, potassium, sodium and albumin. All patient results of these tests processed during six months were taken from the laboratory information system. Using the MA Generator software, different MA procedures were analysed. Different inclusion criteria, calculation formulas, batch sizes and weighting factors were tested. Selection of optimal MA procedures was based on their ability to detect simulated biases of different sizes. After optimization, the validation of MA procedures was done. The results were presented by bias detection curves and MA validation charts.

**Results:**

Simple MA procedures for albumin and sodium without truncation limits were selected as optimal. Exponentially weighted MA procedures were found optimal for creatinine and potassium, with the upper truncation limits of 150 μmol/L and 6 mmol/L, respectively.

**Conclusions:**

It has been experimentally confirmed that it is possible to perform the selection, optimization and validation of MA procedures using the bias detection simulation method. Also, it is possible to define MA procedures optimal for a laboratory with a small daily testing volume.

## Introduction

Analytical quality control is one of the key tasks facing biochemists in a medical laboratory. For this purpose, internal quality control materials are analysed in certain time intervals and laboratories participate in external quality control programs (*1*, *2*). But, in order to prevent releasing erroneous patient results if an error occurs between two control measurements, there is a need for developing quality control plans based on risk management (*3*). In this light, the use of patient samples for the purpose of continuous quality control can be considered through calculating the moving average (*4*). Moving average (MA) means calculating the average value from a set of patient results and further using that value for control purposes. It is called “moving” because the MA is recalculated every time a new result is received, which means data is continuously updated and evaluated as patient samples are analysed. The most commonly used algorithms for calculating MA values are: simple MA, exponentially weighted moving average (EWMA) and Bull’s algorithm (*5*). Simple MA is the non-weighted average value of a selected number of consecutive results. Exponentially weighted moving average is a formula for calculating an average value which includes previous measurements modified with an exponential weighting factor. The weighting for each piece of data is changed exponentially, by adjusting the “importance” assigned to more recent measurements compared to the older. The smaller the weighting factor, the less importance is assigned to the new results and *vice versa*. Bull’s algorithm is a complex formula that calculates an average value from batches of 20 measurements on a haematologic analyser. The idea of a MA was presented by Hoffman and Waid in 1965, suggesting a follow-up of the average of normals (AON) for control purpose (*6*). In 1974, Bull *et al.* published a study on various methods for defining MA control procedures based on the results of patients’ erythrocyte indexes (*7*). In 1984, Cembrowski and colleagues defined guidelines for the implementation of the “average of normals” procedures for quality control in medical laboratories (*8*). However, although it has been implemented in almost all haematologic analysers in the form of Bull’s algorithm, the MA has never been widely applied in medical laboratories for several reasons. One is the complexity of defining optimal MA procedures, which are specific to each laboratory and therefore cannot be generalized or downloaded from any other source, but require individual selection, optimization and validation (*4*). The second reason limiting the application of MA procedures is the lack of insight in the ability of selected MA procedures to detect a clinically significant bias. In particular, there is the question of their ability to detect the occurrence of bias for less frequently ordered tests and in laboratories with a small daily number of samples and performed tests (*1*). In recent years, researchers’ interest in this topic has been on the rise again, with new proposals for ways in which MA procedures could be optimized for routine use (*1*, *2*). One of them is the MA bias detection simulation method proposed by Van Rossum *et al.* (*9*). They described in detail the use of bias detection curves and MA validation charts (*10*).

The aim of this study was to apply the method for the selection, optimization and validation of MA procedures described by Van Rossum in a laboratory with a small daily testing volume and to select the MA procedures that can be used as an additional tool for analytical quality control (*9*, *10*).

## Materials and methods

### Materials

The study was conducted at the Department of laboratory diagnostics, Railway Healthcare Institute, using data from the laboratory information system Next lab (BitImpex, Belgrade, Serbia). Four analytes were chosen for the study: creatinine, potassium, sodium and albumin. The selection was based on the daily number of tests performed. Creatinine was chosen as a representative of the high frequency tests, sodium and potassium as moderately frequent and albumin as a low frequency test in our laboratory. All the results of these parameters obtained over a period of six months (January-June 2018) were extracted from the laboratory information system (LIS). During this time, both internal and external quality controls were within acceptable limits for all four analytes. Numerical values of the patient results, as well as the exact measurement times (date, hour, minute, second) were extracted, without clinical or demographic data. The results were taken from the LIS with a preserved sequence of measurements on the analyser, which enabled the dataset to keep the within-day and day-to-day variations. All results were obtained from adult outpatients because our Institute takes care of the general adult population at the primary healthcare level without any department of specific pathology. All tests were performed on a clinical chemistry analyser Architect c16000 (Abbott, Abbott Park, USA), with the original reagents. The use of LIS data for the purpose of this study was approved by the Ethical Committee of the Railway Healthcare Institute.

### Methods

For each of the 4 examined analytes, a selection of inclusion criteria (truncation limit), calculation formula (simple MA or EWMA), size of the batch or the weighting factor (depending on the formula) and control limits were made. The only inclusion criteria used were truncation limits. Truncation limits are the end points of concentration ranges of examined analytes which are included in MA calculation. Values outside of these limits are outliers and they are excluded from calculation. The choice of truncation limits depends on the patient population with which the laboratory is working. Based on spread in the concentration values of examined analytes, the following truncation limits were tested: for creatinine, upper limit 150, 200, 300 and 400 μmol/L; for sodium, lower limit 130 mmol/L, upper limit 145 mmol/L; for potassium, upper limit 6 mmol/L; for albumin, lower limit 30 g/L. For each of the 4 analytes, calculations were carried out first without the inclusion criteria, and then with them.

For the calculation of MA values, 2 formulas were used: simple MA and EWMA. Both simple MA and EWMA formula were examined for each analyte. Simple MA is calculated using the formula: z _(t)_ = x _(t)_ / n + x _(t-1)_ / n + x _(t-2)_ / n + ... + x _(t- n + 1)_ / n, where z _(t)_ is the calculated mean value on the result number t, x is the result, n is the batch size. The size of the batch is the number of consecutive test results which are used for calculating a MA value in a simple MA algorithm. Each time a new result is received, MA is recalculated from the number of results that is configured as the batch size. The following batch sizes were used: 5, 10, 25, 50 and 100 results. Exponentially weighted moving average is calculated using the formula: z _(t)_ = λ x _(t)_ + (1-λ) z _(t-1)_, where z _(t)_ is the calculated mean value on the result number t, x is the result and λ is the weighting factor. As the starting point for z_(t-1)_ the mean of the overall population was used. For each new result, new MA value was recalculated. Weighting factor is a coefficient which determines how much the current and the previous test results affect the calculation in the EWMA algorithm. It can take values between 0 and 1. Based on the literature data the following weighting factors were used: 0.2, 0.1, 0.05 and 0.02 (9).

Control limits are MA values which, when exceeded, trigger a MA alarm. In order to establish the upper and lower control limits of an MA procedure, the maximum and minimum value of the MA were used. By choosing control limits to be the maximum and minimum value of the MA, we should almost completely avoid generating false alarms when optimized MA procedures are put into routine work. These minimum and maximum values were obtained for each combination of calculation formula and truncation limits. Also, they were calculated without truncation limits.

Bias simulation was performed for every 400 consecutive results. The following bias quantities were introduced into the results of all 4 analytes: ± 50%, ± 40%, ±30%, ±20%, ± 10%, ± 5%, ± 3%, ± 1%. In addition, for each analyte, a bias equal to the allowable total error (TEa) for that test was additionally introduced. This was done considering TEa as clinically significant bias. The Clinical Laboratory Improvement Amendments (CLIA) data were used for TEa, because they were already used in our laboratory for Sigma metrics calculations (*11*). Values were rounded to the nearest integer value, since the software does not allow the use of decimal numbers. So, the introduced additional biases were: for creatinine ± 15%, for potassium ± 18% (TEa 17.97%) and for sodium ± 4% (TEa 3.57%). For albumin, TEa coincided with an already tested bias of ± 10%.

The obtained results were presented and analysed using MA bias detection curves and MA validation charts. For each investigated combination of the calculation formula and the size of the batch with a simple MA or the value of a weighting factor in EWMA, an appropriate bias detection curve was constructed. The curves were constructed using graphics on which the size of the introduced bias is expressed as percent on the x axis. On the y axis is the number of results necessary to detect a certain bias with the examined MA procedure. Multiple candidate curves were constructed on the same graph, representing different MA procedures for one analyte, to allow a visual comparison of the different MA procedures. This visual assessment was the basis for selecting all the elements of an optimal MA procedure: truncation limits, calculation formula, batch size or weighting factor and control limits.

Validation of the selected MA procedures for each of the examined analytes was done using validation charts. Validation charts are bar diagrams in which the size of a bias is shown on the x axis, and the number of results needed for bias detection on the y axis. The columns represent the median of the required number of results, and the error bars are the minimum and maximum numbers, thus providing a detailed insight into the ability of bias detection for each examined procedure. Median number of results required to detect a certain bias indicates that in 50% of cases, the bias will be detected in less than that number of results, and in 50% of cases in more than that. Error bars give us data on the minimum and maximum number of results in which a certain bias will certainly be detected, from which we can conclude whether this is possible within our daily production or not, which was the basis of our optimization strategy.

### Statistical analysis

The normality of distribution of the analysed data was tested by the Shapiro-Wilk test using the statistical software IBM SPSS Statistics, version 25.0 (IBM Corp, Armonk, NY, USA). All MA calculations and simulations were performed using MA Generator software (Huvaros B.V., Bloemendaal, The Netherlands).

## Results

The characteristics of the analysed datasets (number of results used for MA calculations, daily testing volumes and obtained concentrations) are shown in Table 1.

**Table 1:** Characteristics of analysed datasets

**Analyte**	**Total number** **of results**	**Average daily** **number of results**	**Median (Q1-Q3) concentration**	**Minimum concentration**	**Maximum concentration**
Creatinine	14,800	121	70 (63-80)	26	971
Potassium	7299	60	4.3 (4.1-4.6)	2.8	7.4
Sodium	6663	55	140 (139-141)	123	149
Albumin	2408	20	43 (41-44)	20	52
Q1-Q3 - interquartile ranges. Concentrations are expressed in μmol/L for creatinine, in mmol/L for potassium and sodium and in g/L for albumin.					

### Comparison of different MA procedures

**Figure 1:**
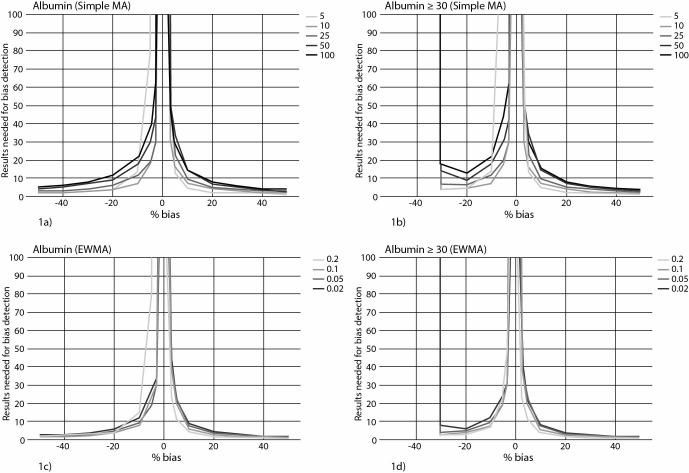
Comparison of multiple moving average curves for albumin. Curves represent the median number of results required for bias detection. Various shades of gray show moving average (MA) procedures with different sizes of the batch (5, 10, 25, 50 and 100 results) for calculating a simple MA or with different weighting factors (0.2, 0.1, 0.05 and 0.02) for calculating EWMA. On figures 1a and 1c, MA procedures without truncation limits are shown and on figures 1b and 1d, the same procedures but with a lower truncation limit of 30 g/L. EWMA - exponentially weighted moving average.

Multiple candidate bias detection curves were constructed on the same graph, representing different MA procedures for one analyte. This allowed a visual comparison of the different MA procedures, as shown for albumin in Figure 1. From the four presented graphs in Figure 1, it can be seen that simple MA calculation for albumin had better performance than EWMA. Also, MA procedures for albumin without truncation limits were better than those with truncation limits, because the introduction of a lower truncation limit of 30 g/L did not improve the detection of small biases, and disabled the detection of negative biases greater than 30%.

**Figure 2:**
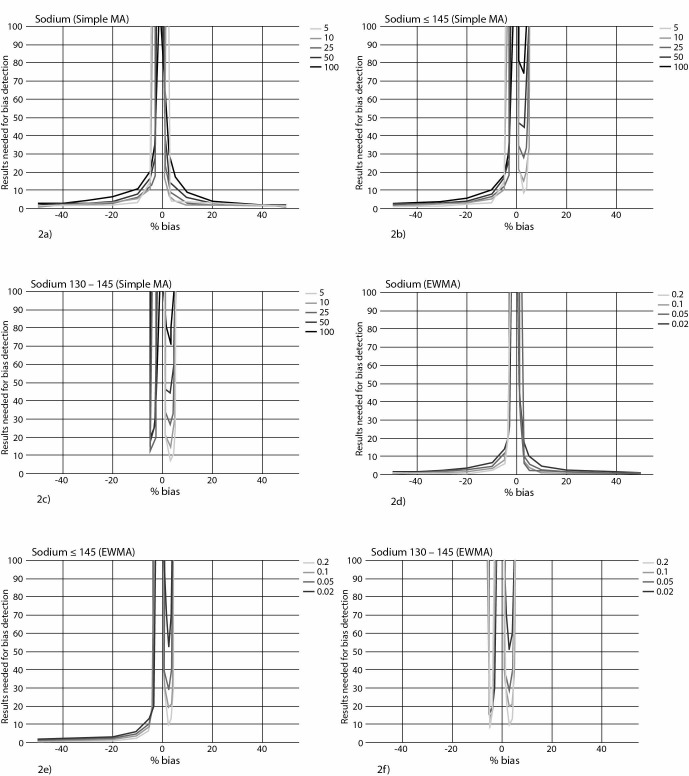
Influence of truncation limits on moving average curves for sodium. 2a) Moving average (MA) curves for sodium at different sizes of the batches for simple MA, without truncation limits. 2b) Curves of the same MA procedures for sodium after the introduction of an upper truncation limit (≤ 145 mmol/L). 2c) Curves of the same MA procedures for sodium, with lower and upper truncation limits (130 mmol/L ≤ Na ≤ 145 mmol/L). 2d) MA curves for sodium at different weighting factors for EWMA, without truncation limits. 2e) Curves of the same EWMA procedures for sodium after the introduction of an upper truncation limit (≤ 145 mmol/L). 2f) Curves of the same EWMA procedures for sodium, with lower and upper truncation limits (130 mmol/L ≤ Na ≤ 145 mmol/L). EWMA - exponentially weighted moving average.

Similarly, as shown in Figure 2 for sodium, introduction of an upper truncation limit of 145 mmol/L led to an insignificant improvement in the detection of small biases for sodium, while substantially compromising the detection of positive biases greater than 5% (Figure 2b). The introduction of a lower truncation limit of 130 mmol/L disabled the detection of negative biases larger than 5% (Figure 2c). Simple MA procedures without truncation limits proved to be the best for sodium (Figure 2a).

**Figure 3:**
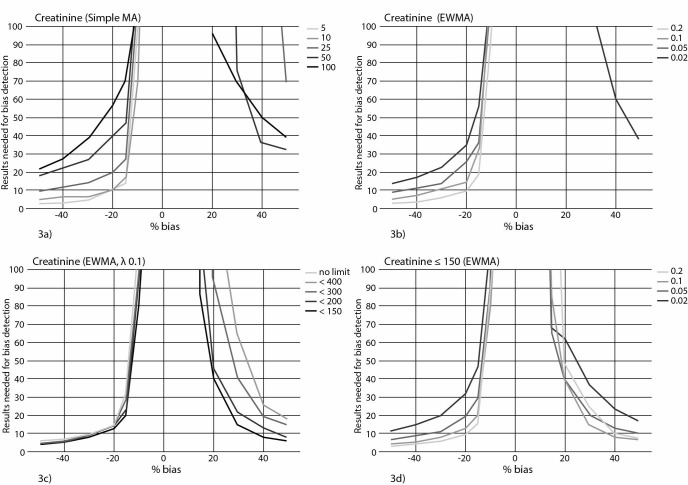
Moving average curves characteristics for creatinine. Various colours show moving average (MA) procedures with different sizes of the batch (5, 10, 25, 50 and 100 results) for calculating a simple MA without a truncation limit (3a) or with different weighting factors (0.2, 0.1, 0.05 and 0.02) for calculating EWMA without truncation limits (3b). Influence of different truncation limits (no limit, values ≤ 400 μmol/L, 300 μmol/L, 200 μmol/L and 150 μmol/L) on MA procedures for creatinine (EWMA algorithm, weighting factor 0.1) (3c). Influence of different weighting factors on MA procedure (EWMA algorithm, upper truncation limit 150 μmol/L) (3d). EWMA - exponentially weighted moving average.

In contrast, the introduction of a truncation limit for creatinine improved the performance of the MA procedure, especially when it came to positive biases. The truncation limit of 150 μmol/L was shown to be optimal for creatinine (Figure 3). Comparison of bias detection curves for different calculation formulas and weighting factors showed that EWMA algorithm had advantages over simple MA for creatinine and that weighting factor 0.1 was optimal for this calculation (Figure 3d).

**Figure 4:**
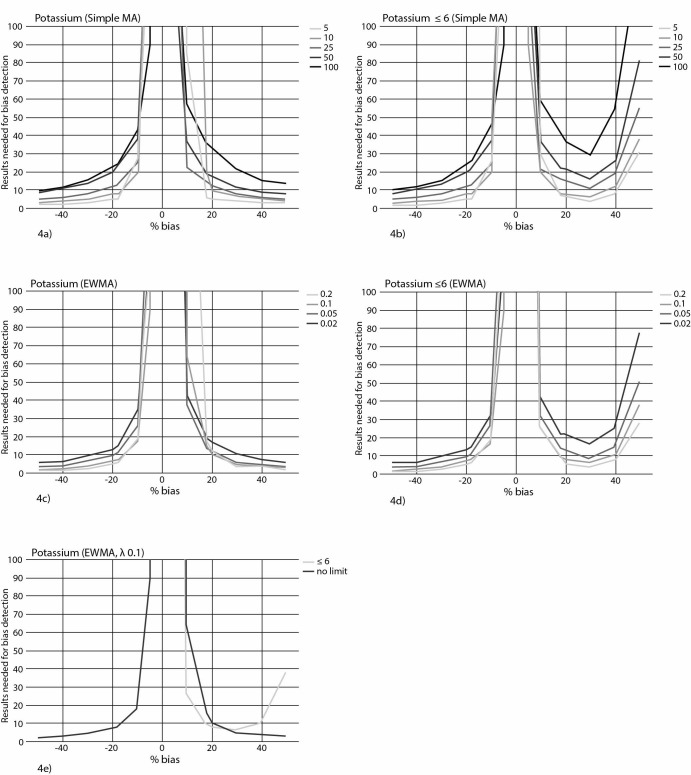
Bias detection curves for moving average procedures for potassium. Numbers in the figure legends represent the batch size for simple MA (4a, 4b), weighting factors for EWMA (4c, 4d) and truncation limit (4e). Truncation limit for potassium is expressed in mmol/L. EWMA - exponentially weighted moving average.

For potassium, a comparison of two candidate curves on the same graph (Figure 4) showed that the MA procedure with an upper truncation limit of 6 mmol/L had better performance in detecting positive biases, while for negative biases the curves completely coincided, that is, the characteristics in detecting negative biases were identical.

### MA procedure validation

**Figure 5:**
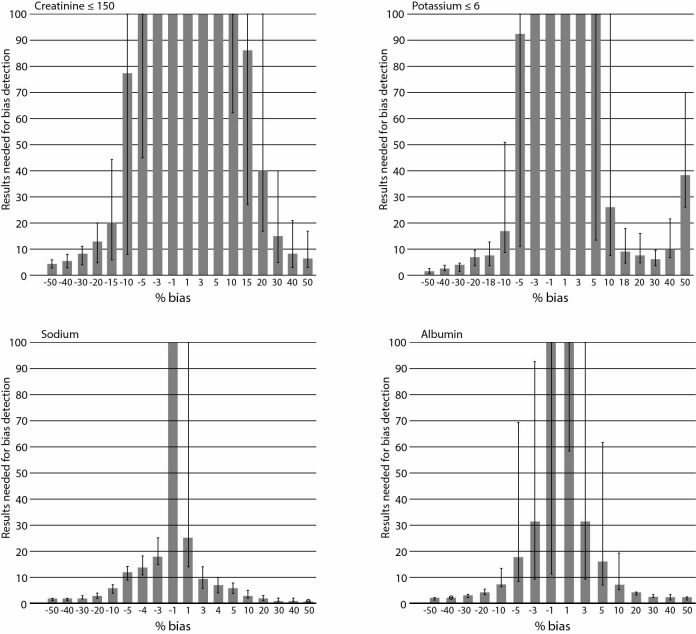
Moving average validation charts for selected optimal MA procedures for creatinine, potassium, sodium and albumin. For each size of the bias, the median, minimum and maximum number of results required for bias detection can be read. Bars represent median and error bars minimum and maximum numbers. Truncation limits for creatinine and potassium are expressed in mmol/L.

Using validation charts, for each selected MA procedure, the details of its ability to detect bias were established (Figure 5). Furthermore, it was possible to precisely determine the bias detection details for two MA procedures between which differences had already been detected in the bias detection curve, as was the case with potassium (Figure 4e, 5). The selected MA procedure for potassium is able to detect bias the size of TEa 18% slightly faster and smaller biases between 10 and 18% much faster. In this case, the detection of positive biases greater than 40% is slower, but it is still possible within the number of 60 potassium tests, which is the daily average in our laboratory.

**Figure 6:**
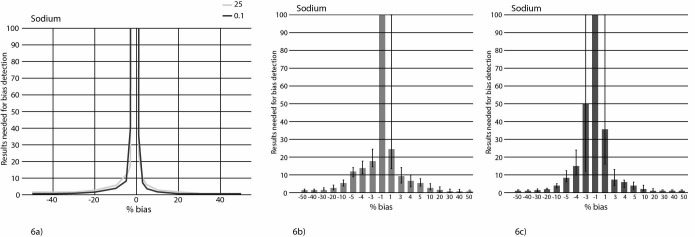
6a) Candidate curves for the optimal moving average (MA) procedure for sodium: simple MA with a batch size of 25 and EWMA with a weighting factor 0.1. 6b) MA validation chart for simple MA for sodium with batch size 25. 6c) MA validation chart for the EWMA with a weighting factor 0.1. EWMA - exponentially weighted moving average.

Also, using validation charts, it was possible to definitively choose between two candidate procedures with very similar bias detection curves, as shown for sodium in Figure 6.

### Selection of optimal MA procedures

Taking into account all of the foregoing, one optimal MA procedure for each of the four examined analytes was selected. The characteristics of these optimal MA procedures are shown in Table 2.

**Table 2:** Number of results (median, minimum and maximum) needed to detect a bias equal to the allowable total error by selected MA procedure for each of the four tested tests

**Analyte**	**Average daily** **number of results**	**TEa (%)**	**Median**	**Minimum**	**Maximum**
Creatinine	121	- 15	20	6	44
		+ 15	86	27	360
Potassium	60	- 18	8	4	13
		+ 18	9	5	18
Sodium	55	- 4	14	11	18
		+ 4	7	4	10
Albumin	20	- 10	7	6	13
		+ 10	7	5	19
TEa – allowable total error. Clinical Laboratory Improvement Amendments data for TEa were used.					

Based on the reading from MA validation charts, we have established MA procedures with which we will be able to detect a clinically significant bias in one fifth to one quarter of the daily potassium production, in one fifth to one third of the daily sodium results, and within the daily production of albumin, despite the very low ordering frequency of this test. For creatinine, a negative clinically significant bias will certainly be detected in one third of the daily production, while positive bias detection within two-thirds of the daily number of results is certain in about 50% of cases. Ability of selected MA procedures to detect a clinically significant bias is shown in Table 3.

**Table 3:** Calculation algorithm, truncation limits and control limits of MA procedures selected as optimal for each of the 4 analytes

	**Calculation algorithm**	**Truncation limit**		**Control limit**	
		lower	upper	lower	upper
Creatinine	EWMA Weighting factor: 0.1	/	150	62	90
Potassium	EWMA Weighting factor: 0.1	/	6.0	3.9	4.8
Sodium	Simple MA Batch size: 25	/	/	137	142
Albumin	Simple MA Batch size: 10	/	/	40	46
MA - moving average. EWMA - exponentially weighted moving average. The values are expressed in μmol/L for creatinine, in mmol/L for potassium and sodium and in g/L for albumin.					

## Discussion

The novelty of our study is that it showed, for the first time in available literature, that optimization of MA procedures is possible in laboratories with low daily testing volume. Also, we found out that by using MA procedures, clinically significant bias can be detected even on less frequent tests.

Our study highlighted the advantages of applying MA procedures in a primary healthcare laboratory. The characteristics of our patient population have resolved some difficulties other researchers have encountered. Firstly, there was no need to separate the results of outpatients and inpatients (*2*). Also, there is a noticeable difference in the median, as well as in the minimum and maximum values of the analysed datasets in our work, compared to other researchers, because the values in the population which our laboratory works with are homogeneous (*10*). In addition, there was no adverse effect of the weekend on the MA procedure such as in a hospital setting, where extreme values mostly appear on the weekends (*4*, *8*). All these reasons are in agreement with other authors’ observations that in primary care laboratories, MA procedures may exhibit better performances than in hospitals (*9*).

When choosing the parameters of a calculation algorithm, our most important findings were related to weighting factors or batch size and truncation limits. We have shown that neither low weighting factors in EWMA, nor large series in the simple MA, are always the best option, as indicated by power function analysis. It is true that a calculating algorithm with a small weighting factor improves the rapid detection of small biases, but then more results are required to detect large biases (*10*). For both tests in which we selected the EWMA procedure (creatinine and potassium), the optimal factor was 0.1, the second largest of the four tested. Also, although in the case of simple MA batch sizes up to 100 samples were examined; series of 10 samples for albumin and 25 for sodium were optimal. We confirmed that constant batch size cannot be defined as a universal model (*8*). Given the low biological variation of sodium and albumin, it is expected that smaller batches will be shown as optimal, while biomarkers with higher biological variation, such as iron or bilirubin, would probably require a higher number of results in the series (*12*).

When it comes to inclusion criteria, unlike the power function analysis and in agreement with Van Rossum’s results, we have found that setting a truncation limit can delay the detection of large biases – an upper limit the detection of large positive, and a lower limit the detection of large negative biases – due to the exclusion of a certain number of results from MA calculations (*8*, *9*). In our case, in a population that does not have a lot of extreme values; the best bias detection is achieved with the widest limits. But, in hospital settings, unlike us, Van Rossum and associates obtained an optimal MA procedure for albumin with a lower inclusion criterion of 20 g/L, and for sodium with a lower inclusion criterion of 125 mmol/L and an upper limit of 150 mmol/L. However, in each population, as well as in the general population coming to laboratories at the primary healthcare level, like ours, there are some outliers. Due to their presence, setting a truncation limit gives better performance for some tests, and so, as we have shown, optimal procedures for creatinine and potassium are those with truncation limits (*8*).

Since there is no precise definition of an optimal MA procedure, we have done optimization with the intention of using the MA as an additional tool for continuous quality control that would allow monitoring of the analytical process between two measurements of control samples (*10*). Therefore, the first criterion for us was to establish an MA procedure for each analyte that would be capable of detecting a clinically significant bias within the daily number of tests, which would otherwise not be detected till the next morning, or until the next measurement of the control sample. Establishing such procedures should allow us to develop a quality control plan that will not require additional measurements of commercial control samples, even in the case of tests with low sigma values (*13*). We considered allowable total error (TEa) as clinically significant bias. The second criterion we managed in the optimization was that the MA procedure was capable of detecting all biases greater than the TEa, since all of them, of course, are clinically significant (*13*, *14*). By combining these two criteria, we made individual compromises for each analyte. If two procedures were approximately equally good in detecting bias the size of the TEa, but one dramatically lost the ability to detect larger biases, we would quit it. On the other hand, where it was possible to find an MA procedure that would quickly detect bias the size of the TEa and smaller, while the detection of larger biases, although delayed, remained possible within the daily production of our laboratory, such a procedure was given advantage. A key tool in determining these details about the ability of MA procedures to detect certain biases were the MA validation charts offering statistically clear data. This practice differs from the “simulated annealing” method, which uses an average number of patient samples affected until error detection (ANPed parameter) based on the mean, not the median (*2*). In order for continuous MA control to make sense, the number of patient results needed to detect a clinically significant bias should not be greater than the number of samples to be analysed between two QC measurements (*1*). This issue is particularly important for less frequent tests, as albumin in our study. From all of the above, it is clear that the selection of MA procedures for each test requires much fine tuning and cannot in any way be a general rule (*8*).

One more important thing shown in our study is the necessity of using a MA method for whose optimization and validation dedicated software is available. In the past decades, visual evaluation and power function analysis were used to optimize and validate MA procedures (*7*, *8*). Recently, Sampson *et al*. have described CUSUM logistic regression to establish control procedures based on patient results (*1*). Ng *et al.* have presented the “simulated annealing algorithm” that introduces the concept of ANPed (*2*). But among all these methods, we decided to use the bias detection simulation method described by Van Rossum for two reasons (*9*, *10*). The first is that it provides easily understandable graphical presentation of the ability of an MA procedure to detect the overall bias. The other reason is the availability of dedicated software (MA Generator). This software enabled us to quickly and easily perform calculations with a large number of variations of the formula, batch size, weighting factor, truncation limits, number of simulations, size of the bias, place where the bias is introduced (*15*).

In addition, we wanted to repeat in our laboratory, in an almost identical manner, an MA experiment already published by another author, bearing in mind that the crisis of reproducibility in medical research is increasingly being discussed today, even related to the analytical performance of routine laboratory tests (*16*). Believing that for research advancement and for the practical application of research results in routine laboratory work, it is necessary to be convinced of the reproducibility of the research, we decided to repeat the experiment on the same tests and method described by the Dutch authors but in our laboratory setting (*9*, *10*, *17*).

Regarding the limitations of our work, the first one is the fact that software simulation and optimization of MA procedures have been performed on a historical set of laboratory results. Their value has not been verified in real time, and their contribution to the overall quality control assurance in our laboratory has yet to be shown. The other limitation to be mentioned is the definition of TEa as a clinically significant bias. Information on the TEa can be obtained from multiple sources. To date, there has been no standardization or harmonization of these different sources, and there is no consensus on what goals to use (*18*). Believing that each laboratory should select objectives that are practical and achievable for each test, for the purposes of this study, we used the CLIA data for TEa (*11*, *19*). One more limitation to be declared is the fact that we didn’t use filters when extracting potassium results from LIS, such as increased haemolysis index or high platelet count. Preanalytical quality procedures in our laboratory include rejection of results based on the haemolysis index and we will take into consideration defining additional filters in LIS in our future MA studies.

In conclusion, we have successfully applied the bias detection simulation method to find the optimal MA procedure in a laboratory with a small daily testing volume using bias detection curves and validation charts, previously described by Van Rossum *et al.* (*9*, *10*). We showed that it is possible to define MA procedures that are optimal for detecting a clinically significant bias. Further research should be directed to the implementation of optimized MA procedures in LIS and examination of their characteristics as continuous quality control in real time.
